# P-377. The Clinical and Economic Burden of Diagnosed or Reported Surgical Site Infections in Procedures Under Surveillance by the National Healthcare Safety Network

**DOI:** 10.1093/ofid/ofae631.578

**Published:** 2025-01-29

**Authors:** ChinEn Ai, Molly Jung, Samantha Bastow, Ghislene N Adjaoute, David L Bostick, Kalvin Yu

**Affiliations:** BD - Becton, Dickinson and Company, Franklin Lakes, New Jersey; BD - Becton, Dickinson and Company, Franklin Lakes, New Jersey; BD, Franklin Lakes, New Jersey, United States of America, Franklin Lakes, New Jersey; BD, Franklin Lakes, New Jersey, United States of America, Franklin Lakes, New Jersey; BD, Franklin Lakes, New Jersey, United States of America, Franklin Lakes, New Jersey; Becton, Dickinson and Company (BD), Franklin Lakes, New Jersey

## Abstract

**Background:**

The attributable costs of surgical site infections (SSIs) have relied on diagnosis codes which have limitations. Moreover, the association between SSI with hospital-onset bacteremia and fungemia (HOB), a proposed quality metric, is not known. We quantified the attributable burden of SSI using two definitions with contemporary data.

**Figure:**
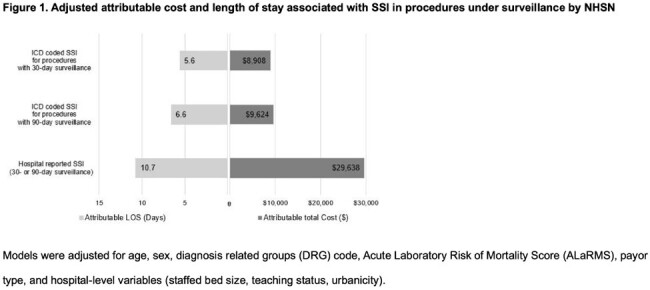

**Methods:**

We conducted a cross-sectional analysis using data from 85 hospitals within the BD Insights and Research Database from October 2015 through June 2019. Two definitions of SSI were considered. The first definition was ICD coded SSI with a National Healthcare Safety Network (NHSN) reportable procedure. The alternative definition was hospital-reported SSI that was identified by an infection preventionist and reported to the NHSN. HOB was defined as a positive blood culture collected within the hospital-onset period (on or after day 4 of hospitalization) for an eligible blood stream infection organism as defined by the NHSN. We estimated and compared key burden metrics - hospital cost, length of stay (LOS), and HOB – in hospitalizations with and without SSI and procedures under NHSN surveillance.

**Figure:**
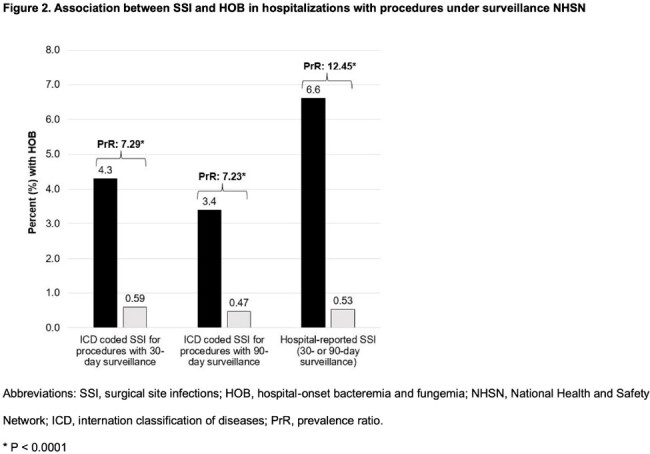

**Results:**

There were 222,298 hospitalizations with a NHSN reportable procedure. The rate of ICD coded SSI under surveillance for 30-days and 90-days was 1.6 and 1.5 per 100 admissions, respectively. Hospital-reported SSI occurred in 0.17 per 100 admissions with a reportable procedure. SSI admissions compared to hospitalizations with the NHSN surveillance procedure without SSI was associated with statistically significant higher cost (attributable cost of ICD coded SSI: $8,908 for 30-days and $9,624 for 90-days; hospital-reported SSI: $29,638, P< 0.0001; Figure 1) and LOS (attributable LOS of ICD coded SSI: 5.6 days for 30-days and 6.6 days for 90-days; hospital-reported SSI: 10.7 days, P< 0.0001).

HOB occurred in 3.4-6.6% of hospitalizations with SSI during the procedure surveillance period. The risk of HOB was 7-to-12-fold higher in procedure admissions with SSI compared to those without (Figure 2).

**Conclusion:**

Regardless of how SSI was identified, SSI was associated with higher cost, longer LOS, and a high burden of HOB. SSIs may also be a novel risk factor for HOB, which should be considered in future quality programs.

**Disclosures:**

**ChinEn Ai, MPH**, Becton, Dickinson and Company: BD employee|Becton, Dickinson and Company: Stocks/Bonds (Public Company) **Molly Jung, MPH**, Becton, Dickinson and Company: BD employee|Becton, Dickinson and Company: Stocks/Bonds (Public Company) **Samantha Bastow, PharmD, MBA**, Becton, Dickinson and Company: BD employee|Becton, Dickinson and Company: Stocks/Bonds (Public Company) **Ghislene N. Adjaoute, MSBA, MSW**, Becton, Dickinson and Company: BD employee|Becton, Dickinson and Company: Stocks/Bonds (Public Company) **David L. Bostick, PhD**, Becton, Dickinson and Company: BD employee|Becton, Dickinson and Company: Stocks/Bonds (Public Company) **Kalvin Yu, MD, FIDSA**, Becton, Dickinson and Company: BD employee|Becton, Dickinson and Company: Stocks/Bonds (Public Company)

